# Expression of ATP-sensitive potassium channels in human pregnant myometrium

**DOI:** 10.1186/1477-7827-9-35

**Published:** 2011-03-21

**Authors:** Chen Xu, Xingji You, Lu Gao, Lanmei Zhang, Rong Hu, Ning Hui, David M Olson, Xin Ni

**Affiliations:** 1 Department of Physiology, Second Military Medical University, Shanghai 200433, China; 2 Department of Gynecology and Obstetric, Navy General Hospital, Beijing 10037, China; 3 Department of Gynecology and Obstetric, Changhai Hospital, Second Military Medical University, Shanghai 200433, China; 4 Departments of Obstetrics and Gynecology, Pediatrics and Physiology, University of Alberta, Edmonton T6G 2 S, Canada

## Abstract

**Background:**

Potassium channels play critical roles in the regulation of cell membrane potential, which is central to the excitability of myometrium. The ATP-sensitive potassium (KATP) channel is one of the most abundant potassium channels in myometrium. The objectives of this study were to investigate the protein expression of KATP channel in human myometrium and determine the levels of KATP channel in lower and upper segmental myometrium before and after onset of labour.

**Methods:**

Both lower segmental (LS) and upper segmental (US) myometrial biopsies were collected at cesarean section from pregnant women not-in-labour (TNL) or in-labour (TL) at term. Protein expression level and cellular localization of four KATP channel subunits in US and LS myometrium were determined by Western blot analysis and immunohistochemistry, respectively. The contractile activity of myometrial strip was measured under isometric conditions.

**Results:**

Four KATP channel subunits, namely Kir6.1, Kir6.2, SUR1 and SUR2B were identified in pregnant myometrium. While found in vascular myocytes, these subunits appear to be preferentially expressed in myometrial myocytes. Diazoxide, a KATP channel opener, inhibited the spontaneous contractility of pregnant myometrium, suggesting that the KATP channels are functional in human pregnant myometrium. Diazoxide was less potent in TL strips than that in TNL strips. Interestingly, expression of SUR1 was greater in TL than TNL tissues, although no differences were found for SUR2B in these two tissues. For both lower and upper segmental myometrium, Kir6.1 and Kir6.2 were less in TL compared with TNL tissues.

**Conclusions:**

Functional KATP channels are expressed in human pregnant myometrium. Down-regulation of Kir6.1 and Kir6.2 expression in myometrium may contribute to the enhanced uterine contractility associated with the onset of labour.

## Background

Human myometrium undergoes dramatic physiological and biochemical changes during pregnancy and parturition. Myometrium remains in a relatively quiescent state during most time of pregnancy, but it develops the highly organized and powerful contraction with onset of labour [1]. The molecular mechanisms underlying the transit of myometrium from a state of relative quiescence to the activated and contractile state are not fully understood. However, it has been proposed that this process is associated with coordinated expression of various proteins including the receptors of uterotonic and uterorelaxant factors, GAP junction and ion channels [1-3].

Potassium channels play critical roles in the regulation of cell membrane potential, which is central to the excitability and contractility of myometrium [4,5]. The opening of these channels results in K^+ ^efflux, causing the membrane potential to closer to K^+ ^equilibrium potential, and thereby reducing excitability and contractility of the smooth muscle cells. Thus, the changes in the expression or activity of K^+ ^channels can translate into a change in excitability and contractility of myometrium.

The ATP-sensitive potassium (K_ATP_) channel is one of the most abundant potassium channels and likely contributes to the resting membrane potential in smooth muscle tissues [6]. The channel comprises heteromultimers of an inwardly rectifying K^+ ^channel (Kir) and a modulatory sulphonylurea receptor subunit (SUR) which is responsible for the ATP sensitivity and pharmacological properties [7-10]. Functional studies indicated that K_ATP _channel plays a role in the regulation of myometrial activity during pregnancy [11-13]. The K_ATP _channel mRNA has been identified in pregnant myometrium. Chien et al [14] had shown the transcripts of K_ATP _channel subunits in pregnant rat myometrium. Curley and coworkers detected the mRNA expression of Kir 6.1, Kir 6.2, SUR1 and SUR2B in human pregnant myometrium and demonstrated that Kir6.1/SUR2B may be the predominant isoform of K_ATP _channel in human myometrium [15]. However, no information is available on the protein expression of K_ATP _channel subunits in myometrium.

It seems that there is a functional regionalization in human myometrium during pregnancy and labour. The upper segment (US) region of the uterus maintains a relaxatory phenotype to accommodate the growing fetus throughout most of gestation and then at labour contracts to cause expulsion of the fetus, while the lower segment (LS) region is supposed to display a contractile phenotype during most time of pregnancy and at onset of labour transforms into a relaxatory phenotype, thereby allowing passage of fetus [16]. Current studies regarding the expression of K_ATP _channel in human myometrium are restricted to LS [15]. There is a lack of literature addressing the changes in the expression of K_ATP _channel in the different region of uterus during pregnancy and labour. Exploring this issue will expand our knowledge regarding the mechanisms controlling human parturition.

The objectives of study are to confirm the expression of K_ATP _channel subunits at protein level in human pregnant myometrium and examine whether the expression of these proteins in different region of uterus is changed during labour.

## Methods

### Tissue collection

The lower uterine segmental myometrial tissues from pregnant women were collected in Changhai Hospital, the affiliated hospital of Second Military Medical University. Upper uterine segmental myometrial tissues from pregnant women were collected in Navy General Hospital, the teaching hospital of Second Military Medical University. Approval of this study was granted by human ethic committee of Changhai Hospital, Navy General Hospital as well as human ethic committee of Second Military Medical University. Written informed consent was obtained from each participant.

LS myometrial biopsies were collected at cesarean section from the pregnant women at term pregnancy (37-42 wk) prior to the onset of labour (TNL, n = 13) or during labour (TL, n = 13). Paired US and LS tissues were collected from the pregnant women at term pregnancy (37-42 wk) before the onset of labour (TNL, n = 7) or during labour (TL, n = 4). Labour was defined as regular contractions (< 5 min apart) and cervical dilation (> 3 cm) without oxytocin or prostaglandin administration. Indications for cesarean section included breech presentation, placenta previa, previous cesarean section, cephalopelvic disproportion, failure of labour to progress, fetal distress, or maternal request. Women who had the evidence of underlying disease, such as hypertension, diabetes, preeclampsia, intrauterine growth restriction, etc, were not included in this study. LS uterine samples were removed from the upper margin of the uterine incision after delivery of the fetus and placenta. US samples were taken just below the fundus through the upper incision by using biopsy forceps. Collected samples were then frozen immediately in liquid nitrogen and stored at -80°C. For immunohistochemical analysis, the biopsies were placed in 10% phosphate buffered formalin. Tissues for contractility study were immediately placed in phosphate-buffered saline on ice and transported to the laboratory.

### Immunohistochemistry

Immunohistochemistry was carried out as described previously [17,18]. Briefly, paraffin sections (5 μm) were cut, rehydrated and microwaved in citric acid buffer to retrieve antigens. Immunohistochemistry were performed with the Histostain-SP kit (Zymed, San Franscisco, CA), which uses a biotinylated second antibody, a horseradish peroxidase-streptavidin conjugate, and a substrate-chormogen mixture to demonstrate antigen in the tissue. The specific antibodies for Kir 6.1 (sc-11224), Kir 6.2 (sc-11228), SUR1 (sc-5789) and SUR2B (sc-5793) were purchased from Santa Cruz Biotechnology (Santa Cruz Biotechnology, Inc. Santa Cruz, CA). The tissue sections were incubated with 3% H_2_O_2 _to inhibit endogenous peroxidases and then incubated with 10% rabbit serum for 30 min to block nonspecific antibody binding. The tissue sections were then incubated with the specific antibodies (1:500) for 24 hr at 4 C. The bound antibodies were detected with the biotin-streptavidin-peroxidase system (UltraSensitive-SP-kit, MaiXin Biotechnology, Fuzhou, China) using diaminobenzidine (Sigma-Aldrich) as chromogen. Counterstaining was performed with hemalum. Mouse antihuman smooth muscle α-actin-specific monoclonal antibody (Dako Inc. Carpinteria, CA) was used to stained the smooth muscle cells. Negative controls were performed by substituting primary antibody with a normal serum in same dilution as well as preabsorption of the primary antibody with a ten-fold excess of the blocking peptides (Kir 6.1: sc-11224 P; kir6.2:sc-11228 P; SUR1: sc-5789 P; SUR2B: sc-5793 P).

### Isometric recording of myometrium contraction

The myometrium tissues (about 3 × 3 × 10 mm pieces) were mounted on parallel wires and placed in a 30 ml organ bath filled with Krebs solution maintained at 37°C, bubbled with a gas mixture (95% O_2_-5% CO_2_). The contractile activity was measured isometrically by a tension transducer, followed by computerized recording and processing (MedLab, Nanjing, China). Each strip of myometrium was set up under an initial tension of 1 g and allowed to equilibrate for 90 min, and the Kreb's solution was changed every 30 min. After the regular contractions (regular in frequency and strength) were established, diazoxide (Sigma-Aldrich, St. Louis,MO) was added in a cumulative manner to the bath at 30 min intervals. Appropriate controls (incubation with solvents) were run under similar experimental conditions in rings of uterus obtained from the same woman. Only one concentration-response curve was performed in each uterine strip. The responses were quantified by the amplitude and frequency of the contractions as well as integration of the area under each contractile record (AUC) using software written specifically for this purpose. The AUC was measured from the basal tension over a 10-min period after each stimulus. The effects were evaluated by comparing the experimental responses with the controls (set as 100%). The data of contractility were presented as percentage of control (% of control).

### Western Blot Analysis

Approximately 50 mg of human myometrial tissue was homogenized in ice-cold lysis buffer consisting of 60 mM Tris-HCl, 2% sodium dodecyl sulfate (SDS), 10% sucrose, 2 mM phenylmethylsulfonyl fluoride (Merck, Darmstadt, Germany), 1 mM sodium orthovanadate, 10 μg/ml aprotinin (Bayer, Leverkusen, Germany). Lysates were then quickly ultrasonicated in ice bath, boiled 5 min at 95 C and centrifuged. The supernatants were collected and stored at -80 C. Protein concentrations were measured using a modified Bradford assay. The samples were diluted in sample buffer (250 mM Tris-HCl (pH 6.8), containing 4% SDS, 10% glycerol, 2% β-mercaptoethanol, and 0.002% bromophenol blue) and boiled for another 5 min. Aliquots of proteins were separated by SDS-PAGE (10%) and subsequently transferred to nitrocellulose membranes by electroblotting. The membrane was blocked in 5% skim milk powder in 0.1% Tris-buffered saline/Tween 20 (TBST) at room temperature for 2 h, and then was incubated with antibodies raised against Kir 6.1, Kir6.2, SUR1 and SUR2B (1:500) at 4 C overnight. After another three washes with TBST, the filters were incubated with a secondary horseradish peroxidase-conjugated IgG (1:1000) for 1 h at room temperature and further washed for 30 min with TBST. Immunoreactive proteins were visualized using the enhanced chemiluminescence Western blotting detection system (Santa Cruz). The light-emitting bands were detected with X-ray film. To control sampling errors, the expression of β-actin was also detected. The resulting band intensities were quantitated by using an image scanning densitometer (Furi Technology, Shanghai, China). Peak count values were expressed as densitometric units. The ratio of band intensities to β-actin was obtained to quantify the relative protein expression level of Kir 6.1, Kir6.2, SUR1 or SUR2B.

### Statistical analysis

The data are presented as mean ± SEM. All data were tested for homogeneity of variance by Bartlett's test. The results indicated that the data were normally distributed. Individual comparisons were made by one-way ANOVA followed by LSD-t test. P-value of <0.05 was considered to be significant.

## Results

### Expression and localization of KATP channel subunits in pregnant human myometrium

**Figure 1:**
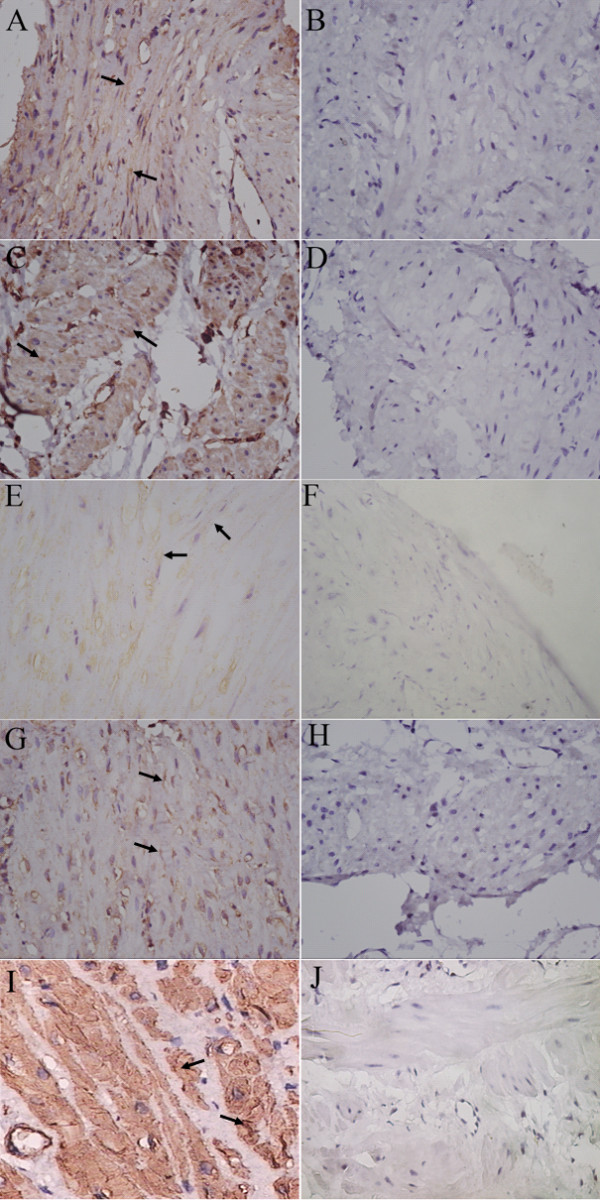
**The expression of K_ATP _channel subunits was identified by immunohistochemistry in pregnant human myometrium**. A, representative section immunostained with the specific antibody against Kir6.1. B, negative control. The primary antibody was substituted by Kir6.1 preabsorption antibody. C, representative section of positive staining for Kir6.2. D, negative control section. The primary antibody was substituted by Kir6.2 preabsorption antibody. E, representative section stained with the specific antibody against SUR1. F, negative control section. The primary antibody substituted by SUR1 preabsorption antibody. G, the section immunostaied with SUR2B antibody. H, negative control section. The primary antibody was substituted by SUR2B preabsorption antibody. I, the section stained with α-actin antibody. J, negative control. The primary antibody was substituted by normal serum. Arrow: positive staining in myometrium smooth muscle cells. Original magnification ×400.

Immunohistochemistry revealed that positive immunoreactivity for Kir6.1, Kir6.2, SUR1 and SUR2B subunit was identified in human pregnant myometrium. These subunits of K_ATP _channel were localized to uterine myocytes. Smooth muscle cells lining blood vessel were also positively stained for these proteins. Immunoreactivity was abolished when the antibody was preabsorbed with excess peptide, thereby confirming the specificity of the antibodies (Figure 1).

### The effect of K_ATP _channel opener on spontaneous contractility of myometrium strip

**Figure 2:**
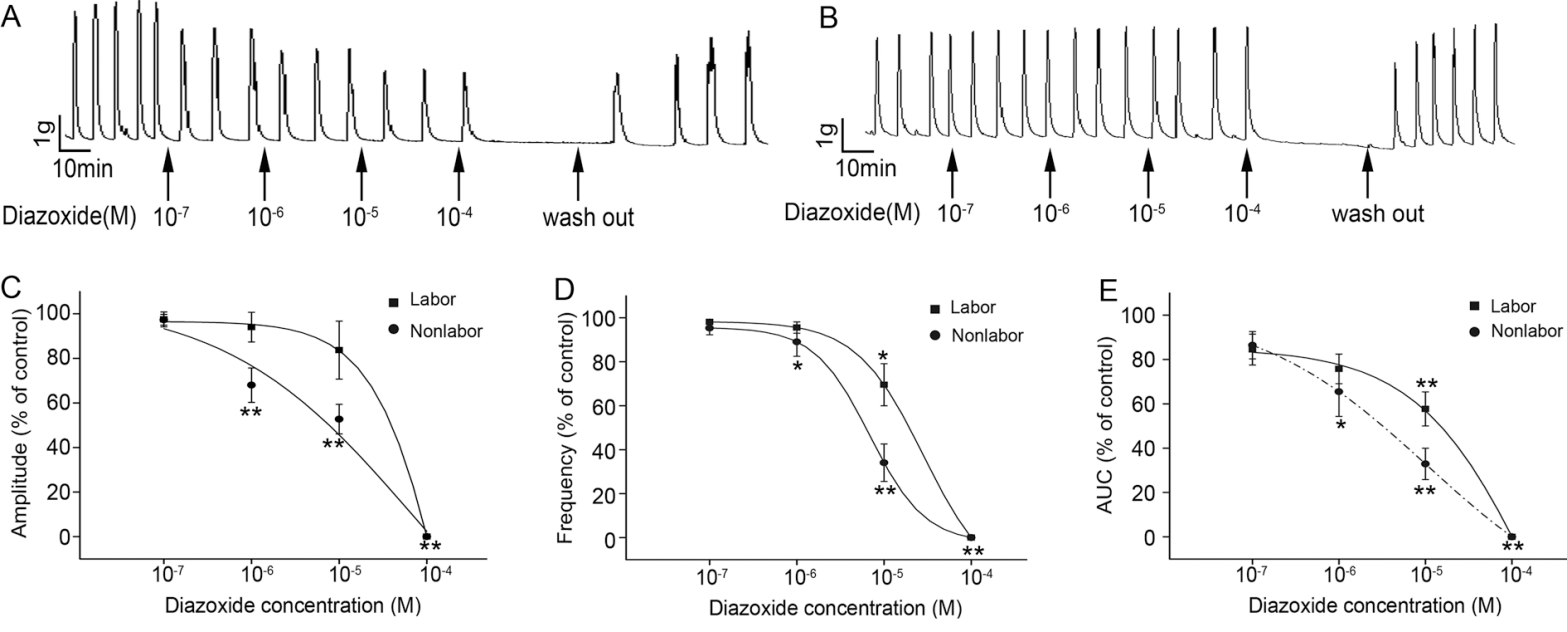
**Effects of diazoxide on spontaneous contractions of human pregnant myometrium**. LS strips were obtained from pregnant women who were not undergoing labour or undergoing labour at term. Myometrial strips were treated with a cumulative increase in concentrations of diazoxide. A&B, representative traces showing the suppressive effect of diazoxide on the spontaneous contractions of TNL strip (A) and TL strip (B). C,D&E, cumulative data of amplitude (C), and frequency (D) and AUC (E) showing the effect of diazoxide on the spontaneous contractility of TNL and TL myometrium.

To confirm the K_ATP _channel activity in pregnant myometrium, we examined the effect of diazoxide, an opener of K_ATP _channels, on spontaneous contractility of LS strips that were obtained from pregnant women who were undergoing labour or not undergoing labour at term. As shown in Figure 2A &2B, treatment of strips with a cumulative increase in concentrations of diazoxide inhibited the phasic contractions. At 10^-4 ^mol/L, diazoxide completely suppressed the spontaneous contractions.

We then compared the effects of diazoxide in nonlabouring and laboring myometrial strips. As shown in Figure 2C-D, the effects of diazoxide on contractility were decreased in TL strips compared with that in TNL strips. The IC50 values of diazoxide in TL group were significantly different from that in TNL group (1.80 × 10^-4 ^± 5.46 × 10^-6 ^mol/L versus 1.06 × 10^-4 ^± 4.37 × 10^-5 ^mol/L for amplitude, *P *< 0.05, n = 3; 3.24 × 10^-5 ^± 2.68 × 10^-5 ^mol/L versus 6.83 × 10^-6 ^± 1.27 × 10^-6 ^mol/L for frequency, *P *< 0.05; n = 3).

### The expression of K_ATP _channel subunits in pregnant myometrium before and during labour

**Figure 3:**
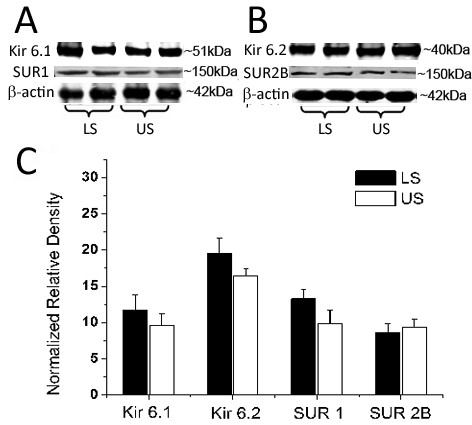
**Expression of K_ATP _subunits in pregnant US and LS myometrium**. Paired US and LS biopsies were obtained from pregnant women at term. The expression level of Kir6.1, Kir6.2, SUR1 and SUR2B were determined by Western blotting as described in Materials and Methods. The values of these subunits from all paired US and LS samples (n = 11) was combined to given an expression profile in the US and LS. A & B, representative protein bands for Kir6.1, SUR1, Kir6.2 and SUR2B. C, cumulative data showing the levels of K_ATP _channel subunits. Data were expressed as mean ± SEM.

Western blotting analysis detected a band of 51 kDa for Kir6.1, 40 kDa for Kir6.2, 150 kDa for SUR1 and 150 kDa for SUR2B in pregnant human myometrium. To give an overall expression profile in the US and LS, the paired expression values from all the patients were combined. When the overall expression level of each protein was compared in the pregnant US and LS samples, there were no significant differences in the levels of each protein between US and LS myometrium (Figure 3).

**Figure 4:**
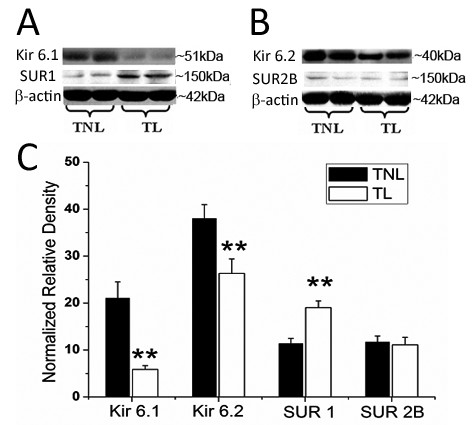
**Semiquantitation of Western blot signals of K_ATP _channel subunits in lower segmental myometrium**. Myometrial tissues were obtained from pregnant women at term before the onset of labour (n = 17) or during active labour (n = 14). A & B, representative protein bands for Kir6.1, SUR1, Kir6.2 and SUR2B. C, cumulative data showing the levels of K_ATP _channel subunits in TNL and TL groups. Data were expressed as mean ± SEM. **P < 0.01 (TL vs TNL).

In lower segmental myometrium samples, the level of Kir6.1 was significantly decreased in TL group compared with TNL group (TL versus TNL, *P *< 0.01). Kir6.2 expression was also significantly down-regulated during labour (TL versus TNL, *P *< 0.05). The expression of SUR1 was increased in TL group compared with TNL group (*P *< 0.01). No marked difference in SUR2B level was observed between TNL and TL groups (Figure 4).

**Figure 5:**
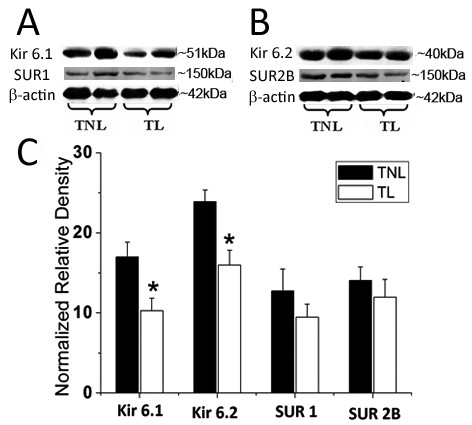
**Semiquantitation of Western blot signals of K_ATP _channel subunits in upper segmental myometrium**. Myometrial tissues were obtained from pregnant women at term before the onset of labour (n = 7) or during labour (n = 4). A & B, representative protein bands for Kir6.1, SUR1, Kir6.2 and SUR2B. C, cumulative data showing the levels of K_ATP _channel subunits in TNL and TL groups. Data were expressed as mean ± SEM. * *P *< 0.05 (TL vs TNL).

In upper segmental biopsies, both of Kir6.1 and Kir6.2 expression were significantly down-regulated in TL group compared with TNL group (TL versus TNL, *P *< 0.05; Figure 5). No significant difference in SUR1 and SUR2B levels was observed between TNL and TL groups.

## Discussion

Some functional studies suggest the K_ATP _channel activity in human myometrium by using the opener and blockers of K_ATP _channels [19-22]. The K_ATP _channels have been identified in human myometrium, but at mRNA level [15]. Considering the fact that not all mRNA is translated to protein due to mRNA instability, it is important to know the protein expression of K_ATP _subunits during pregnancy and labour. The present study demonstrated, for the first time, the protein expression of Kir6.1, Kir6.2, SUR1 and SUR2B subunits of K_ATP _channels in human myometrium and showed the localization of these four subunits in myometrium.

The K_ATP _channel subunits were also identified in smooth muscle layer of uterine vasculature in the pregnant myometrium. Because the proportion of vasculature smooth muscle is very small in the myometrial samples, the protein level of K_ATP _channel subunits can reflect the expression of this channel in uterine smooth muscle.

Different combinations of Kir and SUR isoforms/variants yield tissue-specific K_ATP _channel subtypes with different features and distinct functional properties. For instance, SUR1-Kir6.2 forms the pancreatic β-cell K_ATP _channel [23,24], and SUR2A-Kir6.2 forms the cardiac K_ATP _channel [25]. Two types of smooth muscle-type K_ATP _channels have been cloned and identified, namely Kir6.2-SUR2B channels and Kir6.1-SUR2B channels [10,26]. Some studies have demonstrated the presence of SUR1 subunit in smooth muscle tissues including pig urethra, rat and human myometrium [15,27-29]. Curley and coworkers' study indicated that Kir6.1/SUR2B is the predominant isoforms of K_ATP _channel in human myometrium although they detected the transcript of Kir6.2 and SUR1 [15]. Our present study also found the protein expression of Kir6.1, Kir6.2, SUR1 and SUR2B subunits in human pregnant myometrium. Diazoxide, a K_ATP _opener, is known to activate K_ATP _channels containing SUR1 or SUR2B but not those containing SUR2A [8,10] and has been shown to be able to inhibit the contractility of human myometrium [30]. In the present study, we also found that diazoxide inhibits the spontaneous contractility of human pregnant myometrium. Taken together, our data suggest that subtypes of K_ATP _channel in pregnant human myometrium might be Kir6.1-SUR1, Kir6.1-SUR2B, Kir6.2-SUR2B and Kir6.2-SUR1.

Longo et al [31] reported that a K_ATP _opener inhibited oxytocin-induced contractions in pregnant human myometrium. Sawada et al [32] demonstrated that over-expression of K_ATP _subunits Kir.6.1 and SUR2B contributes to an inhibition of oxytocin-induced uterus contractions in late pregnant rats. The present study found that the relaxatory effects of K_ATP _channel opener were decreased in TL strip compared with TNL myometrium. Thus, it is suggested that a decrease in the expression of K_ATP _channels may facilitate enhanced contractility of the myometrium after onset of parturition.

A number of studies have demonstrated differential expression of contraction-associated proteins (CAPs) such as connexin-43 and prostaglandin receptors in US and LS myometrium after onset of labour [33-35]. However, some studies reported that the expression pattern of some CAPs in US and LS during labour are similar [34,36,37]. Prior studies have shown no differences in contractility between US and LS myometrium [38], which is supported by our own results on the BK channel [17]. In the present study, we found that the modulatory receptor subunit SUR1 was up-regulated in LS, but not in US, after onset of labour, suggesting that the K_ATP _function may differ between LS and US with the onset of labour.

## Conclusions

Our results indicate that K_ATP _channel subunit Kir6.1, Kir6.2, SUR1 and SUR2B are predominately localized to myometrial cells in human pregnant uterus. The down-regulation of K_ATP _channel subunit Kir6.1, Kir6.2 expression in myometrium may contribute to the enhanced uterine contractility associated with onset of labour.

## Competing interests

The authors declare that they have no competing interests.

## Authors' contributions

CX performed Western blot analysis and immunohistochemistry experiments and analyzed the data. XY performed the tension study experiments. LG participated in Western blot and immunohistochemistry experiments as well as data analysis. LZ, RH and NH recruited patients and organized the collection of tissues. DMO participated in manuscript preparation. XN conceived of the study, participated in its design, coordination and wrote the manuscript. All authors read and approved the final manuscript.
